# Respiratory microbiome profiles differ by recent hospitalization and nursing home residence in patients on mechanical ventilation

**DOI:** 10.1186/s12967-020-02642-z

**Published:** 2020-12-07

**Authors:** Min-gyung Baek, Seong Ji Woo, Nam Eun Kim, Chaeyun Baek, Sungho Won, Youngmi Kim, Jae Jun Lee, Hana Yi, Ji Young Hong

**Affiliations:** 1 Department of Public Health, Korea University, Seoul, Republic of Korea; 2 Institute of New Frontier Research, Hallym University College of Medicine, Chuncheon, Republic of Korea; 3 Department of Public Health Science, Graduate School of Public Health, Seoul National University, Seoul, Republic of Korea; 4 Institute of Health and Environment, Seoul National University, Seoul, Republic of Korea; 5 Interdisciplinary Program of Bioinformatics, Seoul National University, Seoul, Republic of Korea; 6 School of Biosystems and Biomedical Sciences, College of Health Science, Korea University, Seoul, Republic of Korea; 7 Division of Pulmonary and Critical Care Medicine, Department of Medicine, Chuncheon Sacred Heart Hospital, Hallym University Medical Center, 77, Sakju-ro, Chuncheon-si, Gangwon-do 200-704 Republic of Korea

**Keywords:** HCAP, Microbiome, Pneumonia, Mechanical ventilation

## Abstract

**Background:**

Healthcare-associated pneumonia (HCAP) is a heterogeneous disease. We redefined nursing-home- and hospital-associated infections (NHAI) group by revising existing HCAP risk factors. The NHAI group comprised nursing home residents with a poor functional status, or recent (past 90 days) hospitalization or recent (past 180 days) antibiotic therapy. Our aim was to determine whether respiratory microbiota profiles are related to newly defined NHAI group in critically ill patients on mechanical ventilation.

**Methods:**

The 180 endotracheal aspirates (ETAs) from 60 mechanically ventilated ICU patients (NHAI group, n = 24; non-NHAI group, n = 36) were prospectively collected on days 1, 3 and 7 in a university hospital. The bacterial community profiles of the ETAs were explored by 16S rRNA gene sequencing. A phylogenetic-tree-based microbiome association test (TMAT), generalized linear mixed models (GLMMs), the Wilcoxon test and the reference frame method were used to analyze the association between microbiome abundance and disease phenotype.

**Results:**

The relative abundance of the genus *Corynebacterium* was significantly higher in the pneumonia than in the non-pneumonia group. The microbiome analysis revealed significantly lower α-diversity in the NHAI group than in the non-NHAI group. In the analysis of β-diversity, the structure of the microbiome also differed significantly between the two groups (weighted UniFrac distance, Adonis, *p* < 0.001). The abundance of *Corynebacterium* was significantly higher, and the relative abundances of *Granulicatella, Staphylococcus, Streptococcus* and *Veillonella* were significantly lower, in the NHAI group than in the non-NHAI group.

**Conclusions:**

The microbiota signature of the ETAs distinguished between patients with and without risk factors for NHAI. The lung microbiome may serve as a therapeutic target for NHAI group.

## Background

Among the recommendations of the 2016 and 2019 American Thoracic Society/Infectious Diseases Society of America (ATS/IDSA) guidelines was the removal of the category of healthcare-associated pneumonia (HCAP) [1, 2]. This is in contrast to the 2005 guidelines, which advocated the use of broad-spectrum antibiotic therapy in HCAP and nosocomial pneumonia [3]. The change was based on several studies showing that, while patient characteristics such as comorbidities and age are determinants of the risk of multidrug-resistant (MDR) infection, this is not the case for all of the HCAP criteria [4–6]. Jones et al. showed that acceptance of the HCAP criteria lowered the threshold for broad-spectrum antibiotics for infection with nosocomial pathogens [7]. However, some HCAP criteria, such as admission from a nursing home or recent (preceding 90 days) hospitalization, are independent predictors of MDR infection [5, 8, 9]. For example, the risk of MDR infections in patients with HCAP requiring mechanical ventilation is similarly high to that of nosocomial infections [10, 11].

Based on previous studies, three risk factors may be better than the previous HCAP criteria for predicting the risk of infection with nosocomial pathogens: recent hospitalization, nursing home residence and antibiotic use in the past 90 days [5, 8, 12]. The application of new, culture-independent methods of characterizing microbial communities, such as 16S rRNA, may lead to better characterization of patient risk groups and more appropriate therapeutic strategies [13]. We hypothesized that the composition of the bacterial microbiome in the respiratory tract during mechanical ventilation would be related to the newly defined risk factors for nursing-home and hospital-associated infections (NHAI), and to the 28-day all-cause and final hospital mortality rates. Therefore, we analyzed the microbial profiles of the endotracheal tube aspirates (ETAs) of intubated patients and determined the relationship between the ETA microbiome and risk factors for NCAI.

## Methods

### Patients and study design

Patients admitted to the intensive care unit (ICU) of Chuncheon Sacred Hospital (South Korea) between July 2017 and October 2018 were prospectively recruited. All patients placed on mechanical ventilation at the time of ICU admission were included in the study. The exclusion criteria were age < 18 years, initiation of mechanical ventilation > 48 h after ICU admission and a duration of mechanical ventilation < 7 days.

In 2005, the ATS/IDSA guidelines introduced HCAP as a new category of pneumonia requiring therapy similar to that prescribed for nosocomial pneumonia, namely broad-spectrum antibiotics [3]. However, several studies showed that HCAP is a heterogeneous disease, and that broad-spectrum antibiotic therapy may not be necessary for all patients with HCAP [6, 8, 14]. Therefore, attempts were made to refine the definition of HCAP based on risk factors for MDR infections. On the basis of the available data [5, 8, 10, 12, 14, 15], we redefined NHAI patients as those meeting at least one of the following criteria: (1) nursing home resident with poor functional status; (2) recent (past 90 days) hospitalization; or (3) recent (past 180 days) antibiotic therapy.

The 60 patients (41 with and 19 without pneumonia) enrolled in this study were divided into NHAI and non-NHAI groups and prospectively followed. This study was approved by the Institutional Review Board of Chuncheon Sacred Heart Hospital (IRB approval number: 2017‐47).

### Data collection and clinical outcome measures

Data on demographic characteristics (age, sex, preexisting comorbidities), the indications for intubation, the presence of acute respiratory distress syndrome (ARDS), the arterial oxygen tension/fraction of inspired oxygen (SaO_2_/FiO_2_) ratio, and the Glasgow Coma Scale (GCS), Acute Physiology and Chronic Health Evaluation (APACHE) II) and Sequential Organ Failure Assessment (SOFA) scores were recorded. The Charlson Comorbidity Index (CCI) score was calculated as described previously [16]. The clinical outcomes of interest in this study were the 28-day all-cause and final hospital mortality rates. Respiratory samples were acquired by endotracheal aspiration, with serial samples collected 1, 3 and 7 days after the initiation of mechanical ventilation.

### DNA extraction, PCR and sequencing

Genomic DNA was extracted using a commercial microbial DNA isolation kit (MP Biomedicals, USA) and then amplified using primers targeting the V3–V4 region of the prokaryotic 16S rRNA gene. The amplicons were purified using XSEP MagBead (CELEMICS), and then subjected to PCR using the Nextera Index Kit (Illumina, USA) following the manufacturer’s instructions. The resultant product was further purified using XSEP MagBead (CELEMICS). The prepared bacterial amplicon library was then quantified, mixed with multiple libraries and sequenced using the MiSeq v3 platform (Illumina).

The raw sequencing data files were preprocessed before downstream data analysis. To remove sequences with low-quality scores, the raw sequence reads were pre-filtered using PRINSEQ [17]. Adapter sequences were removed using CUTADAPT [18]. Paired-end reads were merged using PEAR [19] and then filtered with PRINSEQ. Chimeric sequences and singletons were screened and reduced using USEARCH [20]. Finally, 180 samples were analyzed and an average of 133,219 reads per sample were obtained (minimum, 29,247; maximum, 587,337). The downstream data analysis was carried out using QIIME [21] with the EzBioCloud 16S rRNA gene sequence database [22]. Operational taxonomic units (OTUs) were defined as clusters of sequences with ≥ 97% identity.

### Data processing and statistical analysis

Various Microbial community comparisons were performed using Quantitative Insights in Microbial Ecology software (QIIME, v. 1.8) and the R package Vegan. In the analysis, two period samples (day 1 and day 7) were selected to clarify the differences between groups and to confirm the changes over time. The microbial sequences from the ETAs represented 554 genera and 2009 species. Absolute OTU of each subject for both time points was combined, and species were filtered by two criteria; (1) eliminating strains that appear only in a few patients, (2) removing strains that account for less than 1000 OTU. Finally, 11 predominant genera (*Acinetobacter, Streptococcus, Corynebacterium, Staphylococcus, Prevotella, Neisseria, Veillonella, Mycoplasma, Granulicatella, Actinomyces, Campylobacter*) and 8 predominant species (*Acinetobacter baumannii, Streptococcus mitis, Corynebacterium ulcerans, Staphylococcus caprae, Veillonell adispar, Granulicatella adiacens, Streptococcus parasanguinis, Streptococcus lactarius*) were included in the statistical analysis.

Ace, Chao1, the Shannon index and the Simpson index were used to express α-diversity; β‐diversity (inter-sample diversity) was defined as the extent of the similarity between microbial communities based on the degree of structural overlap. Nonmetric multidimensional scaling plots based on weighted UniFrac distances were used to visualize the differences between groups in microbial community structure. Principle coordinate analysis was conducted using a permutational multivariate ANOVA (PERMANOVA), performed via the Adonis function of the R package vegan (1000 permutations).

We conducted statistical analysis to identify the association of pneumonia and NHAI risk factor on the abundance with each OTU. Each subject was measured at two different time points (day 1 and 7) and generalized linear mixed effects models (GLMM) was utilized to handle the repeatedly observed measurement. Pneumonia and NHAI risk factor were considered as response variables. Relative abundances of OTUs at each time point were Log2 transformed, and its effect was evaluated by including it as a covariate. We also considered the paired and longitudinal distance-based approach (Pldist) to detect the longitudinal changes in the microbiome over time with various outcome types [23]. It can also calculate differences in non-phylogenetic dissimilarities such as Gower’s distance, Bray–Curtis dissimilarities, Jaccard distance and Kulczynski distance between time points. Last we found that pneumonia and NHAI risk factor are time-invariant and thus considered four methods for cross-sectional data; phylogenetic tree-based microbiome association test (TMAT), OMiAT (version 5.1), Wilcoxon test and reference frame method [24, 25]. For the cross-sectional methods, means of log-transformed read count per million (CPM) at two different point (day 1 and 7) were considered as the response variables. Among all approaches, other tests except for reference frame method can adjust the effect of the other covariates and age, sex, APACHE score and CCI were included as covariates for both. GLMM was conducted with SAS 9.4 (Cary, NC), and the other analyses were done with R software. All test results were considered significant using a *p* value of ≤ 0.05.

## Results

### Characteristics of the study participants

The study population consisted of 60 patients who had been placed on mechanical ventilation, including 41 patients diagnosed with pneumonia and 24 who had risk factors for NHAI. Table 1 shows the patient characteristics. The proportions of ARDS, a high C-reactive protein (CRP) level and an abnormal paO_2_/FiO_2_ ratio were higher in patients with than without pneumonia, as expected. Patients in the NHAI and non-NHAI groups did not differ in terms of sex, severity index (APACHE and SOFA) scores, or the GCS score. However, patients in the NHAI group were older and had more comorbidities than the non-NHAI group. There were no significant differences between the two groups in either the 28-day all-cause or final hospital mortality rate.

**Table 1:** Baseline characteristics of the study participants

	Pneumonia	Non-pneumonia	*p* value	NHAI	Non NHAI	*p* value
	n = 41	n = 19		n = 24	n = 36	
Age^a^	73 (61–79)	76 (59–81)	0.744	78 (72–85)	71 (57–77)	0.004
Male^b^	30 (73.2%)	12 (63.2%)	0.431	15 (62.5%)	27 (75.0%)	0.391
ARDS	8 (19.5%)	0 (0%)	0.047	4 (16.7%)	4 (11.1%)	0.702
Pneumonia				23 (95.8%)	19 (52.8%)	< 0.001
Charlson Comorbidity Index^a^	3 (1–4)		0.085	3 (2.3–5.0)	1.5 (0–2.8)	< 0.001
Cause of intubation			< 0.001			< 0.001
Cardiac arrest	1 (2.4%)	3 (15.8%)		0 (0.0%)	4 (11.1%)	
Neurological distress	5 (12.2%)	13 (68.4%)		1 (4.2%)	17 (47.2%)	
Post-operative status	0 (0%)	1 (5.3%)		0 (0.0%)	1 (2.8%)	
Respiratory	35 (85.4%)	2 (10.5%)		23 (95.8%)	14 (38.9%)	
PaO_2_/FiO_2_ ratio	212 (133.5–299)	431 (321–458)	< 0.001	222 (131.3–301.0)	323 (184.3–442.5)	0.022
Severity						
APACHE II score^a^	20 (16–24)	22 (17–25)	0.202	20.5 (16.3–24.0)	21 (17–25)	0.634
SOFA score^a^	7 (6–9)	6 (4–9)	0.117	7 (6–9)	7 (5–9)	0.569
GCS^a^	8 (6–11)	6 (5–9)	0.074	8.5(6.0–10.8)	7.5 (6–9)	0.303
Extubation success in 3 weeks	23 (56.1%)	11 (57.9%)	0.999	14 (58.3%)	20 (55.6%)	0.999
28-day all-cause mortality	12 (29.3%)	6 (31.6%)	0.999	7 (29.2%)	11 (30.6%)	0.999
Final hospital mortality	19 (46.3%)	6 (31.7%)	0.4	11 (45.8%)	14 (38.9%)	0.606
MV duration^a^	13 (8–18)	10 (7–16)	0.335	13 (8–17.5)	10 (7.0–16.8)	0.384
CRP (mg/dl)	133 (45–213.5)	21 (5–132)	0.008	124.5 (62.8–192.0)	94 (8.0–194.0)	0.127

*NHAI* nursing-home- and hospital-associated infections, *ARDS* acute respiratory distress syndrome, *APACHE II* Acute Physiology and Chronic Health Evaluation II, *SOFA* Sequential Organ Failure Assessment, *GCS* Glasgow Coma Scale, *MV* mechanical ventilation, *CRP* C-reactive protein

^a^ Median (interquartile range)

^b^ Frequency (%)

**Fig. 1:**
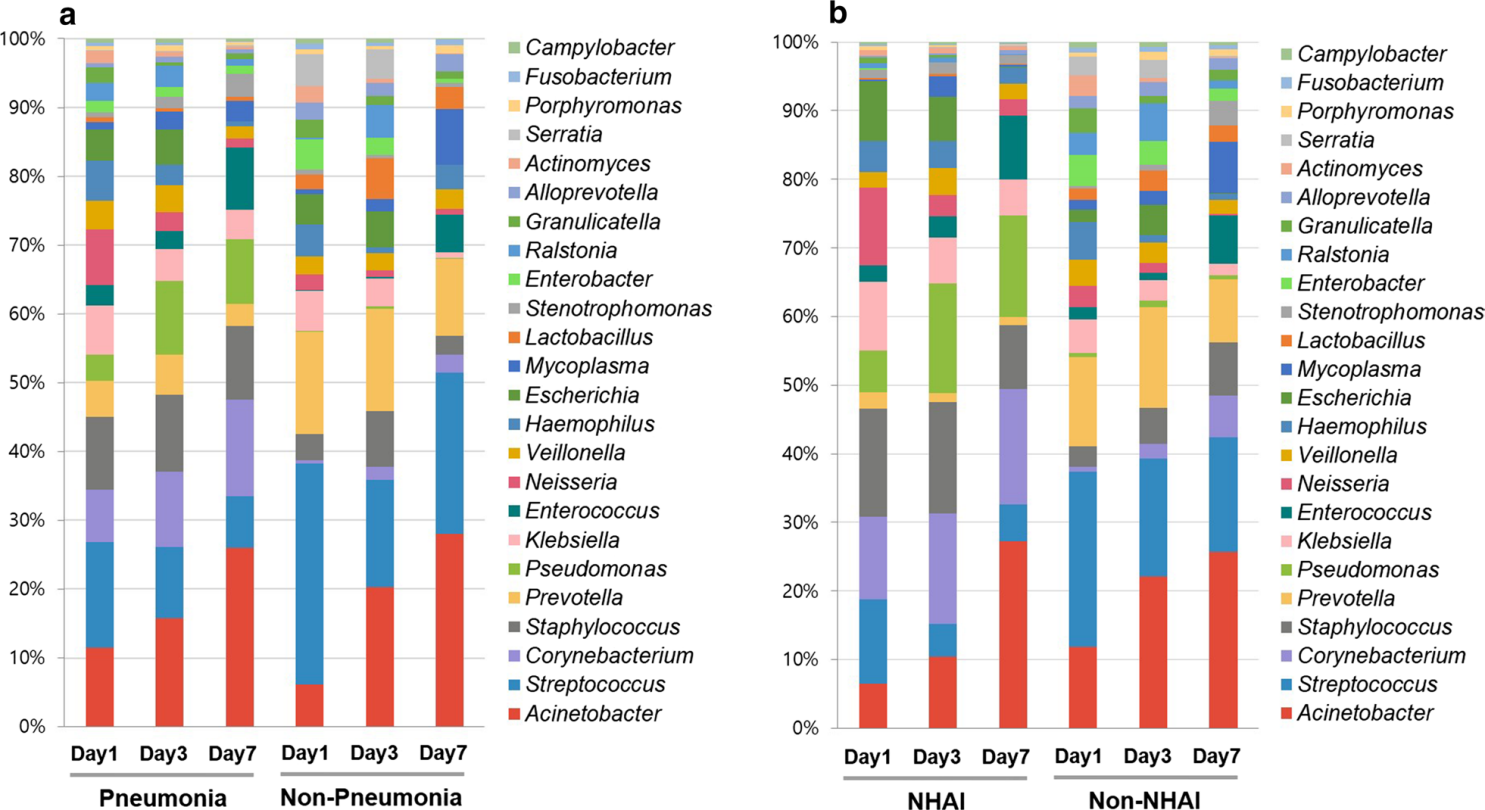
Relative abundance of bacterial communities in the endotracheal aspirates of the study participants over time. Taxonomic summaries of the 180 samples are shown for pneumonia and non-pneumonia patients (**a**) and for NHAI and non-NHAI patients (**b**)

Differences in the microbial composition of the ETAs between patient groups (pneumonia vs non-pneumonia, NHAI vs. non-NHAI) are shown in the taxonomic summary (Fig. 1). The average abundance of the genera *Corynebacterium, Staphylococcus* and *Pseudomonas* was higher in the pneumonia group than in the non-pneumonia group. The relative abundances of *Streptococcus* and *Prevotella* were higher in the non-NHAI group, whereas the relative abundance of *Corynebacterium* was higher in the NHAI group.

### Respiratory microbiota during mechanical ventilation in patients with and without pneumonia

Neither α-diversity nor β-diversity differed significantly between patients with and without pneumonia (not shown). The association between pneumonia and microbial taxa was analyzed, adjusting for the effects of age, sex, and APACHE and CCI scores in the TMAT, optimal microbiome-based association test (OMiAT), and GLMM, but not in the Wilcoxon test. A correlation between *Corynebacterium* and pneumonia was demonstrated by the TMAT and Wilcoxon test (Table 2). Especially, the GLMMs identified a significant difference in the abundance of *C. ulcerans* between patients with and without pneumonia (FDR corrected *p* = 0.035), although it did not find the correlation between *Corynebacterium* and pneumonia at the genus level.

**Table 2:** Bacterial genera associated with pneumonia

Genus	FDR-OMiAT	Beta TMAT	FDR-TMAT	Estimate-RF	FDR-RF	FDR-Wilcoxon	β-GLMM	FDR-GLMM
*Acinetobacter*	0.7832	0.02	0.7321	NA	NA	0.6809	0.014	0.7855
*Corynebacterium*	0.2791	0.1	0.0226	− 2.03	0.9985	0.005	0.102	0.4899
*Granulicatella*	0.7832	− 0.04	0.3564	− 1.89	0.9985	0.3817	− 0.104	0.4899
*Staphylococcus*	0.7832	− 0.01	0.8202	0.09	0.9985	0.9556	0.026	0.725
*Streptococcus*	0.2791	− 0.03	0.7321	0	0.9985	0.3817	− 0.04	0.6638
*Veillonella*	0.7832	− 0.03	0.708	0.35	0.9985	0.6809	− 0.065	0.5494

*FDR* false discovery rate, *OMiAT* optimal microbiome-based association test, *TMAT* phylogenetic-tree-based microbiome association test, *RF* reference frame, *GLMM* generalized linear mixed model

**Fig. 2:**
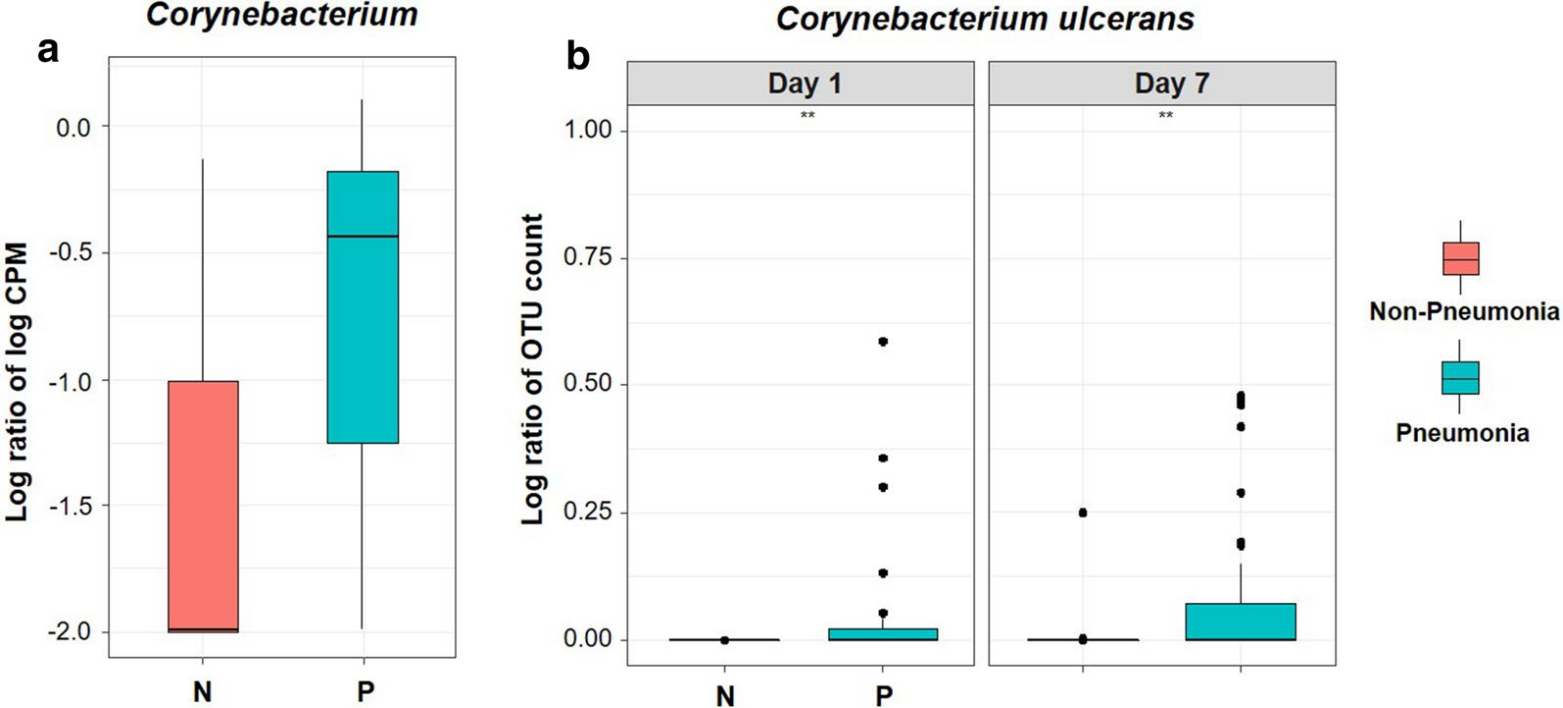
Increased abundance of *Corynebacterium ulcerans* in patients with pneumonia. **a** Box and whisker plot showing the relationship between *Corynebacterium* OTUs and pneumonia group. For the lines in a box and whisker plot: the extreme bars are the overall range, the bottom and top of the box are the 25th and 75th percentiles and the line inside the box is the 50th percentile (median). The significantly increased log-transformed CPM of the genus Corynebacterium in the pneumonia than in the non-pneumonia group was shown by TMAT with false discovery rate using Benjamini–Hochberg correction (FDR corrected *p*-value = 0.0226). **b** Longitudinal patterns of relative abundance of *C*. *ulcerans* OTUs in the pneumonia and non-pneumonia groups. Wilcoxon test was used (Day 1 *p*-value = 0.003, Day 7 *p*-value = 0.007). **p < 0.01

Figure 2a shows the significantly increased log-transformed CPM of the genus Corynebacterium in the pneumonia than in the non-pneumonia group by TMAT with false discovery rate using Benjamini–Hochberg correction. The difference of this genus abundance was mainly caused by a species named *C*. *ulcerans*. No other *Corynebacterium* species detected in the samples showed meaningful difference between the pneumonia and non-pneumonia groups. Figure 2b shows the larger increase in *C*. *ulcerans* in the pneumonia than in the non-pneumonia group, both on day 1 and on day 7 (Day 1 *p*-value = 0.003, Day 7 *p*-value = 0.007). A trend towards a larger difference in the OTU abundance of *Corynebacterium* in the pneumonia group compared to the non-pneumonia group on day 7 was also identified.

### Respiratory microbiota during mechanical ventilation in patients with and without risk factors for NHAI

**Fig. 3:**
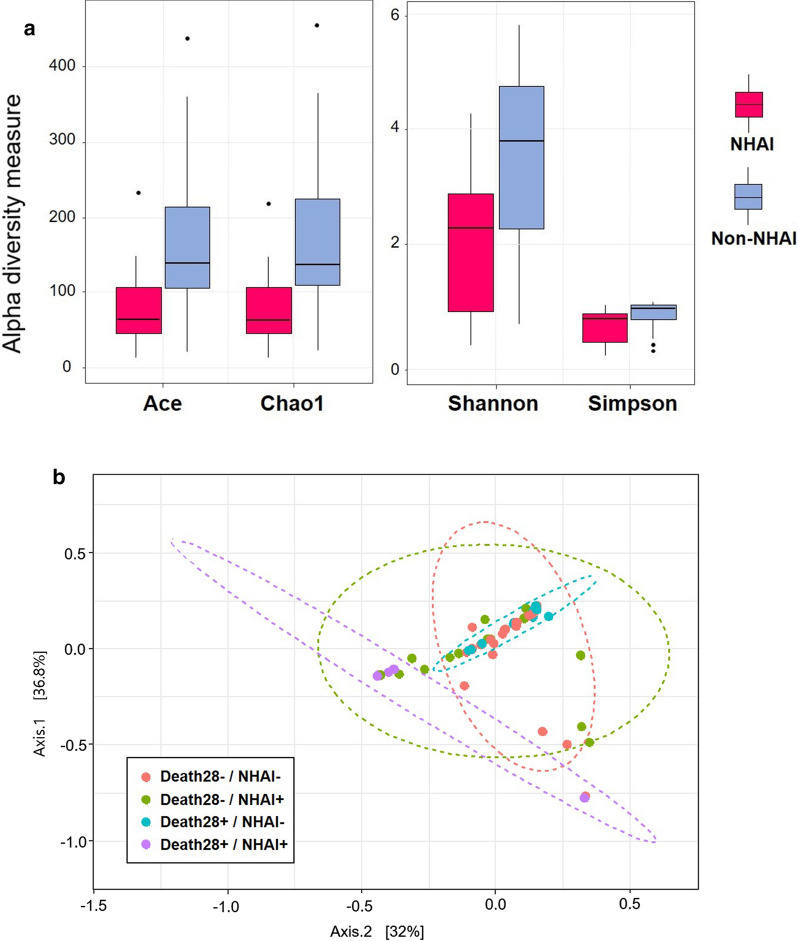
Differences in respiratory microbiome in NHAI group and non-NHAI group. **a** Comparison of the different metrics of α-diversity between the NHAI group (group 1) and non-NHAI group (group 0). For the lines in a box and whisker plot: the extreme bars are the overall range, the bottom and top of the box are the 25th and 75th percentiles and the line inside the box is the 50th percentile (median). **b** Microbial community structure in the endotracheal aspirates according to risk factors for NHAI based on weighted UniFrac distances. A PERMANOVA, performed using the Adonis function in the R package vegan) was conducted to compare the microbial community structure between the NHAI and non-NHAI groups. The x- and y-axis represent the first and second principal coordinates with the proportion of variance. The ellipses represent the 95% confidence interval for each group assuming a multivariate normal distribution. The analysis was adjusted for age, sex, APACHE score and Charlson Comorbidity Index score. ***p < 0.001 ****p < 0.0001

An analysis of α-diversity revealed significant differences between the NHAI and non-NHAI groups (expressed as median [interquartile range]; Fig. 3a). Ace (67.5 [48.2, 108.9] vs. 139.9 [106.2, 215.7]; *p* < 0.001), Chao (64.4 [45.3, 112.0] vs. 137.5 [106.2, 230.6]; *p* < 0.001), Shannon index (2.3 [0.7, 2.9] vs. 3.8 [2.3, 4.7]; *p* < 0.001) and Simpson index (0.7 [0.2, 0.8] vs. 0.9 [0.6, 0.9]; *p* < 0.001) values were lower in the NHAI group than in the non-NHAI group. The microbiome structure of the NHAI group could also be clearly distinguished from that of the non-NHAI group based on the β-diversity (PERMANOVA, adjusted *p* < 0.001, Fig. 3b).

The microbial taxa associated with NHAI were identified using the methods shown in Table 3. Adjustments were made for age, sex, and the APACHE and CCI scores in the TMAT, OMiAT, and GLMMs, but not in the Wilcoxon test. The TMAT and Wilcoxon analyses revealed that *Granulicatella, Streptococcus* and *Veillonella* were negatively correlated with NHAI, while *Corynebacterium* was positively correlated (Table 3).

**Table 3:** Bacterial genera associated with NHAI

Genus	FDR-OMiAT	β-TMAT	FDR-TMAT	Estimate-RF	FDR-RF	FDR-Wilcoxon	Beta-GLMM	FDR-GLMM
*Acinetobacter*	0.5861	− 0.08	0.0807		NA	0.52	− 0.039	0.6165
*Corynebacterium*	0.1788	0.2	0.0004	3.21	0.3302	< 0.05	0.17	0.2166
*Granulicatella*	0.1911	− 0.18	0.0002	− 1.15	0.8524	< 0.05	− 0.318	0.0385
*Staphylococcus*	0.2129	− 0.14	0.023	− 2.22	0.8524	0.344	− 0.142	0.2166
*Streptococcus*	0.2129	− 0.09	0.0132	3.1	0.8524	0.0045	− 0.067	0.4439
*Veillonella*	0.5861	− 0.11	0.0101	− 0.39	0.8611	0.0045	− 0.136	0.2166

*FDR* false discovery rate, *OMiAT* optimal microbiome-based association test, *TMAT* phylogenetic-tree-based microbiome association test, *RF* reference frame, *GLMM* generalized linear mixed model

Similarly, the GLMMs revealed that the genera *Granulicatella* and *Prevotella* were more abundant in the non-NHAI group, while at the species level *C. ulcerans* was more abundant in the NHAI group and *Granulicatella adiacens* was more abundant in the non-NHAI group (Additional file 1: Table S1).

Additional file 2: Figure S1 shows the longitudinal trends in microbial taxa according to the risk factors for NHAI. In ETAs from days 1 and 7, the OTU abundances of *Veillonella, Granulicatella and Streptococcus* were significantly higher in the non-NHAI than in the NHAI group, whereas *Corynebacterium* was more abundant in the

NHAI group. However, none of the longitudinal changes in microbial taxa differed significantly by disease phenotype.

### Longitudinal change in the respiratory microbiome according to clinical outcome

The associations between non-phylogenetic dissimilarities (based on pldist) and the clinical outcomes (the 28-day all-cause and final hospital mortality rates) were analyzed using the microbiome regression-based kernel association test (MiRKAT). The results showed that the variability in the respiratory microbiome was not associated with the clinical outcome (Table 4). The GLMM analysis yielded similar results. There was no association of microbial OTU abundance with the 28-day all-cause or final hospital mortality rate, in either the pneumonia or non-pneumonia group (Additional file 1: Table S2).

**Table 4:** Association between microbiome variability and clinical outcome

	28-day all-cause mortality		Final hospital mortality	
	Species	Genus	Species	Genus
Total (n = 60)				
Bray–Curtis	0.15	0.63	0.45	0.24
Jaccard	0.33	0.57	0.48	0.22
Kulczynski	0.34	0.58	0.42	0.32
Gower	0.29	0.45	0.43	0.23
Pneumonia (n = 41)				
Bray–Curtis	0.3	0.76	0.54	0.36
Jaccard	0.51	0.63	0.43	0.26
Kulczynski	0.5	0.69	0.28	0.3
Gower	0.29	0.63	0.38	0.44
Non-pneumonia (n = 19)				
Bray–Curtis	0.65	0.14	0.65	0.14
Jaccard	0.66	0.5	0.66	0.5
Kulczynski	0.75	0.62	0.75	0.62
Gower	0.71	0.13	0.71	0.13

p values are from the MiRKAT. The quantitative and qualitative analyses (Bray–Curtis, Jaccard, Kulczynski and Gower analyses) were adjusted for sex, age, and APACHE II and CCI scores

## Discussion

Patients with HCAP are a heterogeneous group [14]. Because the existing HCAP criteria did not reflect the risk of nosocomial pathogen infection and led to overuse of broad-spectrum antibiotics, they were omitted from the 2016 hospital-acquired pneumonia/ventilator-associated pneumonia (HAP/VAP) guidelines [1, 7]. Attempts to develop alternative criteria for HCAP, and a specific therapy [8, 11, 14], included our concept of NHAI, which may better predict the occurrence of MDR infections and consequences of continued exposure to hospital and other healthcare environments.

Recent studies using conventional microbiologic culture tests have shown different patterns of infection among countries and geographic regions [26–29]. Studies from the USA and the Asia/Pacific region reported that HCAP is characterized by high frequencies of infections with drug-resistant pathogens [26–28, 30], whereas other studies showed similarities in the involved pathogens between HCAP and community-acquired pneumonia (CAP) [29].

Culture-independent 16S rRNA gene sequencing is useful for detecting fastidious bacteria, the abundance of which tends to be underestimated; this method is more sensitive than conventional culture methods [31]. To our knowledge, this is the first study to use microbiome sequencing to determine the associations of healthcare/hospital-related factors with the composition of the respiratory microbiome of intubated patients.

The most important finding of this study was that the ETA microbiome profile of intubated patients was significantly associated with newly defined risk factors for NHAI. Generally, microbial diversity was higher in patients without than with risk factors for NHAI. The analysis of β-diversity, based on weighted UniFrac distances, showed distinct clusters of samples, identified as NHAI and non-NHAI groups. Beyond microbial diversity, our study showed the association of specific bacterial genera with continued exposure to healthcare and hospital environments.

The abundance of *Corynebacterium* increased, whereas the abundances of *Granulicatella, Streptococcus, Staphylococcus* and *Veillonella* decreased, in intubated patients with risk factors for NHAI. In particular, members of the genus *Corynebacterium* were significantly more abundant in intubated patients with than without pneumonia, and the abundance of *C*. *ulcerans* in the pneumonia group tended to increase over time. Although *Corynebacterium* spp. are among the commensal flora of the nasopharynx [32], a causative role in pneumonia was recently described [33–36]. The identification of *Corynebacteria* subspecies is now possible using new diagnostic technologies, such as matrix-assisted laser desorption ionization time of flight mass spectrometry and 16S rRNA gene sequencing [13, 33–35, 37]. Based on such studies, Clariot et al. suggested that *Corynebacterium* spp. may be responsible for pneumonia in mechanically ventilated patients, similar to our results [33], while Yasuda et al. reported that *C. ulcerans* produces diphtheria toxin and causes severe pneumonia complicated by diffuse pseudomembrane formation in the central airways [36]. *Corynebacterium* spp. may also be responsible for HAP. An analysis of bronchoalveolar lavage fluid (BALF) obtained from Japanese patients revealed *Corynebacterium* spp. in the samples of 11.8% of the patients with HAP, and in 4.9% of those from patients with HCAP; however, *Corynebacterium* spp. was present in only 1.6% of the samples from patients with CAP [37]. Our results also demonstrate the need to consider *Corynebacterium* spp*.* as a possible healthcare/hospital-associated pathogen.

Several studies using molecular techniques have revealed the presence of unexpected microorganisms in specific phenotypes of pneumonia. For example, Steenhuisen et al. found an increase in *Lactobacillus* and *Rothia* in CAP, while Zarkharkina et al. reported a decrease in these microorganisms, in patients with VAP [38, 39]. Similar to our own findings, a study of adult ICU patients identified *Prevotella* as being dominant in controls, whereas *Corynebacteria species* were uniquely present in VAP cohorts [40].

Segal et al. reported that a lung microbiome containing taxa from the oral cavity (i.e., *Streptococcus*, *Veillonella*, *Granulicatella*, and *Prevotella*) induced host cellular mucosal immunity of the Th17/neutrophilic phenotype and blunted TLR4 responses [41]. In the study of Kitsios et al., the dominance of oral taxa within lung communities was strongly associated with culture negativity and weaker host immune responses [42]. From these data, it can be inferred that non-NHAI patients in whom oral taxa dominate the lung microbiome will have a weaker immune response than NHAI patients, which would also explain why patients with HCAP present with more severe disease and have clinical outcomes resembling those of nosocomial pneumonia [26, 28, 43].

Our data showed no difference in microbial community according to clinical outcome; this is in contrast to Dickson et al., who reported that the lung microbiome predicts outcomes in critically ill patients [44]. This discordance may be due to differences in study population and sampling method (ETA vs. BALF).

Our study had several limitations. First, it used a single-center design and the sample size was small. Multicenter studies with larger populations are needed to validate our results. Second, we did not obtain BALF samples, although this was in accordance with ATS guidelines recommending non-invasive testing [1]. Nevertheless, analyzing BAL and ETA samples may enable comparison with the results of other studies, such that more robust conclusions could be drawn. Third, although our findings support the concept of an NHAI risk factor-associated microbiome, its clinical significance should be investigated via functional analyses, for example. Such studies would allow conclusions to be drawn regarding the association between an NHAI-specific microbiome and outcome/immune metabolic responses.

The strengths of our study included the homogeneity of the study population in terms of age (all elderly patients), and the multiple serial samples acquired from each patient. The analysis showed that the composition of the NHAI-related microbiome was similar regardless of antibiotic use or time of day.

## Conclusions

In this prospective observational cohort study of mechanically ventilated patients, the loss of diversity and dysbiosis of the respiratory microbiome were more profound in patients with than without risk factors for NHAI, which were in turn positively associated with the presence of *Corynebacterium*, and negatively associated with that of *Granulicatella, Streptococcus, Staphylococcus* and *Veillonella*. Further studies are needed to determine the clinical relevance of microbial community profiling, and to identify novel therapeutic targets in critically ill elderly patients with risk factors for NHAI.

## Supplementary information

**Additional file 1: Table S1.** Statistics for the relative abundances of bacterial taxa in ETAs of NHAI group based on GLMM analysis. **Table S2.** Association between the relative abundances of the bacterial taxa in the ETAs and clinical outcomes: results from GLMM analyses.

**Additional file 2: Figure S1.** Longitudinal patterns of the relative OTU abundances of the dominant genera in the NHAI and non-NHAI groups. For the lines in a box and whisker plot: the extreme bars are the overall range, the bottom and top of the box are the 25th and 75th percentiles and the line inside the box is the 50th percentile (median). *P < 0.05; **P < 0.01; ***P < 0.001; **** P < 0.0001.

## Data Availability

The datasets used and/or analysed during the current study are available from the corresponding author on reasonable request. We submitted our raw sequence reads to NCBI. The SRA submission is in process and the BioProject accession number is PRJNA678854.

## Abbreviations

HCAP: Healthcare-associated pneumonia
NHAI: Nursing home and hospital associated infection
ICU: Intensive care unit
TMAT: Tree-based microbiome association test
GLMM: Generalized linear mixed effects models
ETA: Endotracheal tube aspirates
OTU: Operational taxonomic units
CAP: Community acquired pneumonia
VAP: Ventilator associated pneumonia
MDR: Multidrug resistant
APACHE II: Acute Physiology, Age, Chronic Health Evaluation II
SOFA: Sequential Organ Failure Assessment
CCI: Charlson comorbidity index
