# Supplementary material for: Respiratory microbiome profiles differ by recent hospitalization and nursing home residence in patients on mechanical ventilation
Source: J Transl Med. 2020 Dec 7;18:464. doi: 10.1186/s12967-020-02642-z (PMC7720271; doi:10.1186/s12967-020-02642-z)
Supplement: Supplementary file 1 — Additional file 1: Table S1. Statistics for the relative abundances of bacterial taxa in ETAs of NHAI group based on GLMM analysis. Table S2. Association between the relative abundances of the bacterial taxa in the ETAs and clinical outcomes: results from GLMM analyses. [file 12967_2020_2642_MOESM1_ESM.docx]

**Additional Tables**

**Table S1. Statistics for the relative abundances of bacterial taxa in ETAs of NHAI group based on GLMM analysis.**

| Taxon |  | β-estimate (FDR *P* value) | | | |
| --- | --- | --- | --- | --- | --- |
|  | **OTU** | **Overall cohort (n=60)** | | **Pneumonia (n=41)** | |
| Genus | *Acinetobacter* | − 0.039 | 0.616 | − 0.117 | 0.432 |
|  | *Streptococcus* | − 0.067 | 0.443 | − 0.006 | 0.948 |
|  | *Corynebacterium* | 0.17 | 0.216 | 0.143 | 0.432 |
|  | *Staphylococcus* | − 0.142 | 0.216 | − 0.25 | 0.207 |
|  | *Prevotella* | − 0.233 | 0.038 | − 0.24 | 0.207 |
|  | *Neisseia* | 0.023 | 0.773 | 0.045 | 0.702 |
|  | *Veillonella* | − 0.136 | 0.216 | − 0.105 | 0.432 |
|  | *Mycoplasma* | − 0.134 | 0.248 | − 0.081 | 0.588 |
|  | *Granulicatella* | − 0.318 | 0.038 | − 0.287 | 0.207 |
|  | *Actinomyces* | − 0.134 | 0.228 | − 0.076 | 0.588 |
|  | *Campylobacter* | − 0.15 | 0.228 | − 0.102 | 0.588 |
| Species | *Acinetobacter_baumannii* | − 0.064 | 0.371 | − 0.121 | 0.371 |
|  | *Streptococcus_mitis* | − 0.153 | 0.215 | − 0.065 | 0.491 |
|  | *Corynebacterium_ulcerans* | 0.293 | 0.018 | 0.22 | 0.115 |
|  | *Staphylococcus_caprae* | − 0.194 | 0.128 | − 0.367 | 0.115 |
|  | *Veillonella_dispar* | − 0.137 | 0.167 | − 0.105 | 0.371 |
|  | *Granulicatella_adiacens* | − 0.336 | 0.018 | − 0.311 | 0.115 |
|  | *Streptococcus_parasanguinis* | − 0.145 | 0.215 | − 0.079 | 0.491 |
|  | *Streptococcus_lactarius* | − 0.153 | 0.215 | − 0.104 | 0.491 |

FDR: false discovery rate

OTU: operational taxonomic unit

**Table S2 Association between the relative abundances of the bacterial taxa in the ETAs and clinical outcomes: results from GLMM analyses.**

|  |  | 28-day all-cause mortality | | | Final hospital mortality | | |
| --- | --- | --- | --- | --- | --- | --- | --- |
| Taxon | Effect | Total | Pneumonia | Non-pneumonia | Total | Pneumonia | Non-pneumonia |
|  | *Acinetobacter* | − 0.024 (0.65) | − 0.064 (0.37) | 0.087 (0.39) | − 0.004 (0.94) | − 0.036 (0.58) | 0.087 (0.39) |
|  | *Actinomyces* | − 0.046 (0.51) | − 0.071 (0.45) | − 0.056 (0.68) | − 0.043 (0.5) | − 0.034 (0.67) | − 0.056 (0.68) |
|  | *Campylobacter* | − 0.045 (0.56) | − 0.046 (0.65) | − 0.117 (0.45) | − 0.023 (0.75) | 0.014 (0.87) | − 0.117 (0.45) |
|  | *Corynebacterium* | 0.065 (0.33) | 0.116 (0.19) | − 0.114 (0.49) | 0.048 (0.45) | 0.063 (0.42) | − 0.114 (0.49) |
| Genus | *Granulicatella* | − 0.03 (0.67) | − 0.055 (0.55) | 0.007 (0.96) | − 0.006 (0.92) | 0.012 (0.88) | 0.007 (0.96) |
|  | *Mycoplasma* | − 0.026(0.71) | 0.026(0.77) | − 0.308(0.14) | − 0.031(0.65) | 0.052(0.55) | − 0.308(0.14) |
|  | *Neisseria* | − 0.066(0.36) | − 0.054(0.53) | − 0.219(0.25) | − 0.006(0.92) | 0.038(0.6) | − 0.219(0.25) |
|  | *Prevotella* | − 0.024(0.68) | − 0.029(0.7) | − 0.073(0.54) | − 0.023(0.67) | 0.009(0.9) | − 0.073(0.54) |
|  | *Staphylococcus* | − 0.05(0.4) | − 0.052(0.45) | − 0.07(0.6) | − 0.049(0.38) | − 0.052(0.43) | − 0.07(0.6) |
|  | *Streptococcus* | − 0.084(0.19) | − 0.121(0.14) | − 0.058(0.63) | − 0.065(0.27) | − 0.073(0.33) | − 0.058(0.63) |
|  | *Veillonella* | − 0.03 (0.61) | − 0.032 (0.66) | − 0.085 (0.54) | − 0.023 (0.68) | 0.002 (0.98) | − 0.085 (0.54) |
| Species | *Granulicatella adiacens* | − 0.029 (0.69) | − 0.053 (0.57) | 0.008 (0.96) | − 0.004 (0.96) | 0.017 (0.84) | 0.008 (0.96) |
|  | *Acinetobacter baumannii* | − 0.044 (0.46) | − 0.09 (0.26) | 0.051 (0.62) | − 0.006 (0.91) | − 0.026 (0.7) | 0.051 (0.62) |
|  | *Staphylococcus caprae* | − 0.03 (0.65) | − 0.024 (0.76) | − 0.069 (0.61) | − 0.03 (0.64) | − 0.016 (0.83) | − 0.069 (0.61) |
|  | *Veillonella dispar* | − 0.036 (0.56) | − 0.035 (0.64) | − 0.106 (0.47) | − 0.025 (0.66) | 0.003 (0.97) | − 0.106 (0.47) |
|  | *Streptococcus lactarius* | − 0.13 (0.17) | − 0.193 (0.15) | − 0.116 (0.49) | − 0.094 (0.26) | − 0.097 (0.35) | − 0.116 (0.49) |
|  | *Streptococcus parasanguinis* | − 0.085 (0.27) | − 0.099 (0.3) | − 0.107 (0.52) | − 0.07 (0.31) | − 0.06 (0.47) | − 0.107 (0.52) |
|  | *Streptococcus mitis* | − 0.077 (0.22) | − 0.105 (0.19) | − 0.063 (0.63) | − 0.055 (0.36) | − 0.054 (0.46) | − 0.063 (0.63) |
|  | *Corynebacterium ulcerans* | 0.061 (0.33) | 0.076 (0.31) | − 0.034 (0.88) | 0.038 (0.53) | 0.018 (0.79) | − 0.034 (0.88) |

Each subject was measured at two different time points and generalized linear mixed effects models (GLMM) was utilized to handle the repeatedly observed measurement.
