# Supplementary material for: Respiratory microbiome profiles differ by recent hospitalization and nursing home residence in patients on mechanical ventilation
Source: J Transl Med. 2020 Dec 7;18:464. doi: 10.1186/s12967-020-02642-z (PMC7720271; doi:10.1186/s12967-020-02642-z)
Supplement: Supplementary file 2 — Additional file 2: Figure S1. Longitudinal patterns of the relative OTU abundances of the dominant genera in the NHAI and non-NHAI groups. For the lines in a box and whisker plot: the extreme bars are the overall range, the bottom and top of the box are the 25th and 75th percentiles and the line inside the box is the 50th percentile (median). *P < 0.05; **P < 0.01; ***P < 0.001; **** P < 0.0001. [file 12967_2020_2642_MOESM2_ESM.docx]

**Figure S1 Longitudinal patterns of the relative OTU abundances of the dominant genera in the NHAI and non-NHAI groups.** **For the lines in a box and whisker plot: the extreme bars are the overall range, the bottom and top of the box are the 25th and 75th percentiles and the line inside the box is the 50th percentile (median).**

**
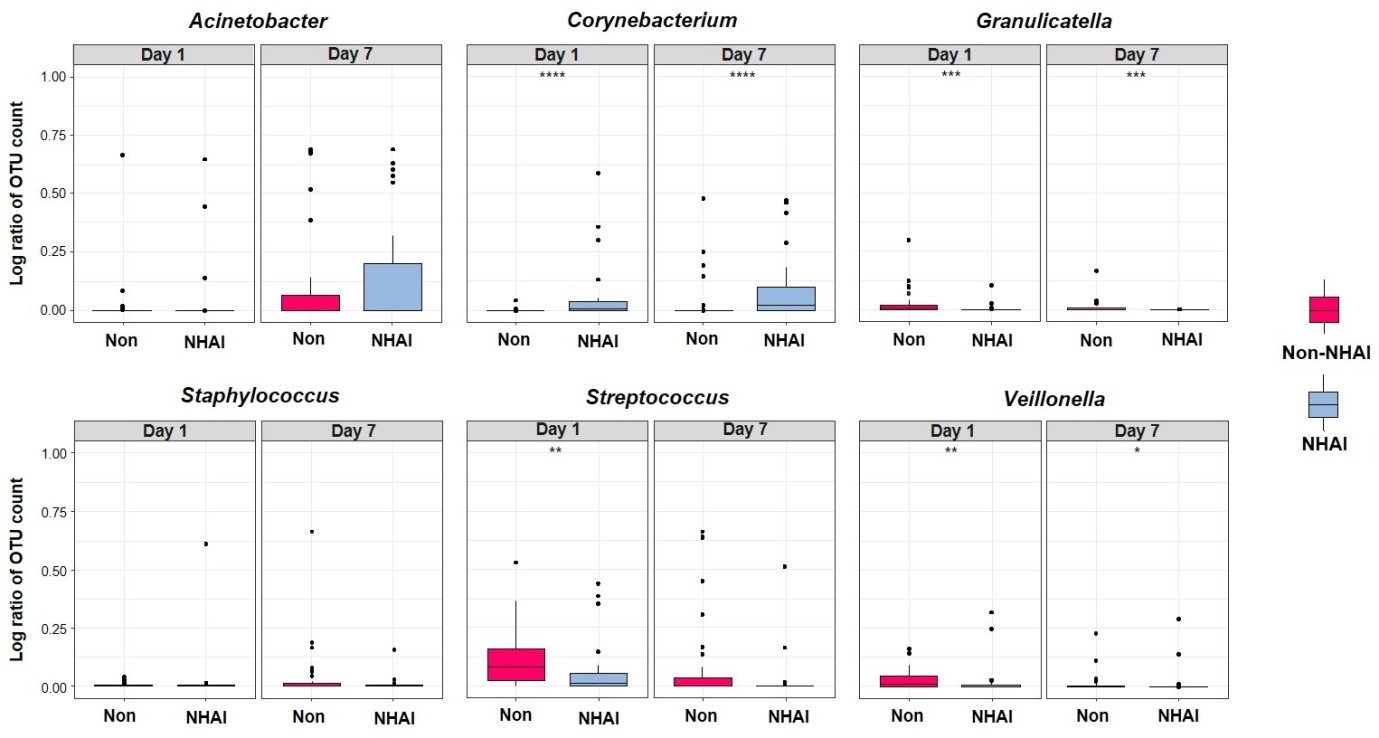
**

*P<0.05

**P<0.01

***P<0.001

**** P<0.0001
